# Pediatric Robotic-Assisted Laparoscopy: A Description of the Principle Procedures

**DOI:** 10.1100/tsw.2006.399

**Published:** 2006-06-21

**Authors:** Carlo Passerotti, Craig A. Peters

**Affiliations:** ^1^ Department of Urology, Children's Hospital Boston, MA, USA; ^2^ Department of Urology, University of Virginia, Charlottesville, USA

**Keywords:** robotic surgery, pyeloplasty, vesicoureteral reflux, child

## Abstract

The advent of clinically useful robotic devices to facilitate reconstructive laparoscopic surgery in pediatric urology opens new doors to minimally invasive procedures. Previously limited by the challenge of delicate suturing and reconstruction using conventional laparoscopic instruments, robotic assistance offers a more rapid climb up the learning curve. Initial procedures that have been safely and efficaciously performed with robotic assistance include nephrectomy, partial nephrectomy, pyeloplasty, and antireflux surgeries. These techniques and their outcomes will be reviewed, as well as some of the challenges still posed by this methodology.

### INTRODUCTION

Surgical robotic systems have evolved rapidly through recent years. Since the first robotic system in urological practice, AESOP, was introduced in 1994, robotic devices have passed through the Zeus system, and the current state-of-the-art is the da Vinci surgical system[1]. With increased precision, efficiency, and better performance, the da Vinci system is beginning to be incorporated into the pediatric urologist's surgical armamentarium.

There are an increasing number of procedures using the da Vinci surgical robotic device. In the pediatric population, ablative renal surgery as well as reconstructive procedures such as pyeloplasty, antireflux procedures, and continent diversion have been reported[2]. With the ability to handle 6-0 and 7-0 suture materials efficiently, the daVinci system is well suited to delicate reconstructive surgery in small children. The system has been enhanced with the addition of a fourth arm to the existing three (endoscope and two working arms), which is best suited to dynamic retraction. The working port size and instruments have been reduced from 8 to 5 mm for pediatric applications.

This article reviews the techniques and results of robotic surgical devices in pediatric urological reconstruction.

## PROCEDURES IN PEDIATRIC POPULATION

### Nephrectomy

Simple nephrectomy was the first major laparoscopic procedure in children and is now well established[3]. The advantage in robotic devices in laparoscopy is the enhanced suturing capability. Simple nephrectomy does not usually need suturing, although it can be useful in place of surgical clips to control the renal vasculature (Fig. 1).

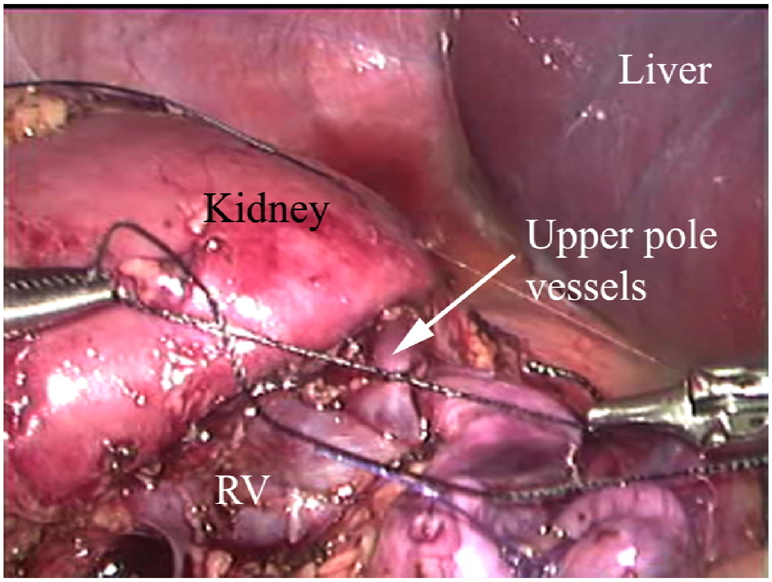

**FIGURE 1**. Suture ligation of upper pole renal vessels during partial nephrectomy. The dexterity of the robotic instruments permits efficient use of suture ligation, which can be more accurate than clip placement in children.

Nephrectomy can be performed by a trans- or retroperitoneal approach, and the transperitoneal operation is the most readily accomplished due to the size of the ports and the arms. For a transperitoneal approach to all renal surgeries, the anesthetized patient is placed supine, secured to the table with an ipsilateral 30° wedge, and the ports are placed in the umbilicus (camera port), midline between the umbilicus and xyphoid, and in the ipsilateral mid-clavicular line below the umbilicus (working ports).

Before the engagement of the robotic system, the patient is turned to 60° and the robot is positioned over the ipsilateral shoulder. The kidney is exposed after mobilizing the colon. The ureter and pelvis are dissected from the lower to the upper pole and the hilum is visualized. The ureter can be used as a handle after transected to expose the hilum. The renal artery and vein are exposed and any branches identified; they are then controlled with surgical clips or free sutures. The remainder of the renal dissection releases the posterior and superior aspects. The kidney is usually removed through the larger port.

The choice of a trans- or retroperitoneal approach depends on the surgeon’s experience and the need for a concurrent additional procedure such as the removal of the entire ureter, management of an intra-abdominal testicle, or contralateral reimplant.

Retroperitoneal access uses different port placement and patient positioning, and is performed in the flank position with the robot. Initial development of the retroperitoneal space is by either a balloon or blunt dissection under direct vision. The hilum is dissected first and the artery and vein controlled before releasing the kidney. With the current robotic system, the space limitations in retroperitoneal renal surgery are a significant factor.

There are no published reports of robotically assisted nephrectomy in children, but our experience has suggested a benefit in older children and if the contralateral ureter requires reimplantation. In the latter case, only four ports are needed, in contrast to two incisions.

### Partial Nephrectomy

Partial nephrectomy can also be performed trans- or retroperitoneally with the daVinci system. Again, due to the size of the ports and the robot arms, the authors prefer to use a transperitoneal approach.

Partial nephrectomy follows similar steps as with a simple nephrectomy regarding port placement and exposure. If the distal ureter needs to be removed, the lower port would be moved closer to the midline. Protecting the vasculature of the remnant pole is the critical challenge in partial nephrectomy, and with the excellent visualization and dexterity of the robotic system, this can be readily done (Fig. 2).

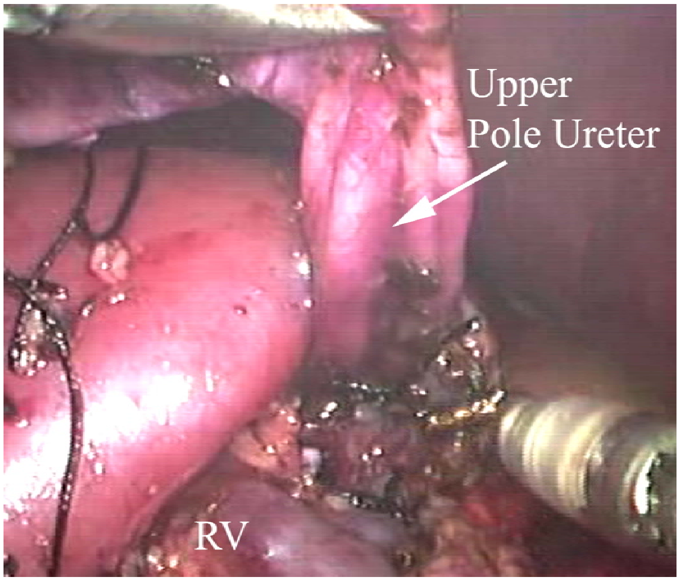

**FIGURE 2.** Development of the plane between the upper and lower poles during a robotically assisted, upper pole, partial nephrectomy for a nonfunctioning, upper pole associated with a ureterocele. The upper pole ureter has been transected, passed beneath the hilar vessles, and is used as a handle to dissect between the poles, avoiding entry into the lower and upper collecting systems. Vascular control of the upper pole has already been achieved.

After vascular control, the abnormal pole is dissected following the delineation between the poles. The affected ureter is also dissected at the level of the lower pole and separated from the remnant ureter, which is protected carefully. Sometimes, the ureteral dissection is made before the hilum is exposed, as the ureter may be useful in mobilizing the affected pole and visualizing the hilum adequately. The poles can be separated using either a cautery or a harmonic scalpel. If the remnant collecting system is entered, it is repaired and a drain is kept in place.

The affected ureter can be removed through this approach. Depending on the presence or absence of obstruction and reflux, the amount of ureter to be removed is determined.

The edge of the normal pole can be sutured with mattress stitches to ensure hemostasis and to limit the risk for leakage. The authors also recommend placing a drain in older children or complex cases. A bladder catheter is kept in place overnight and the patient is discharged when taking oral liquids in sufficient quantity; sometimes, patients prefer the same day.

In a limited number of patients, we have experienced no significant problems with this technique. Operative times are improving. Patients have generally been able to be discharged within 2 days. We have seen one patient with a residual urine collection in the site of the upper pole, an issue that has been reported in laparoscopic partial nephrectomies as well. The enhanced visualization and dexterity with the articulated robotic instruments permits very precise dissection of the upper and lower vasculature, which should enhance safety and efficiency.

### Pyeloplasty

Robotic-assisted laparoscopic pyeloplasty can be performed either trans- or retroperitoneally with the advantage of using smaller suturing as fine as 7-0 with efficiency and accuracy.

For a transperitoneal approach, briefly, the anesthetized patient is placed supine, secured to the table with an ipsilateral 30° wedge, and the ports are placed in the umbilicus (camera port), midline between the umbilicus and xyphoid, and in the ipsilateral mid-clavicular line below the umbilicus (working ports).

Before engaging the robotic system, the patient is turned to 60° up and the robot is brought in over the ipsilateral shoulder. The ureteropelvic junction (UPJ) can be exposed transmesenterically or by reflecting the colon. The procedure follows the same steps as with open technique. The ureter and pelvis are dissected. A hitch stitch is placed through the abdominal wall to lift and stabilize the renal pelvis. The renal pelvis is transected first along the line that will be the future anastomosis. This serves as a handle to permit positioning and spatulation of the ureter without touching the actual anastomotic site. The ureter is spatulated laterally and the anastomosis is performed using running or interrupted 6-0 or 7-0 suture for small children (5-0 for older children). An appropriate-sized double J stent is placed after the posterior wall closure, but can also be placed retrograde if a removal string is requested by the patient or family. The stent is placed through the abdominal wall using an angiocatheter and the guide wire is passed into the ureter. After the placement, the wire is removed and if there is any uncertainty about positioning in the bladder, methylene blue can be instilled and should be seen to reflux into the renal pelvis. The anterior wall anastomosis is the completed. The steps are shown in Fig. 3.

Postoperatively, the Foley catheter is removed in 24 h and the double J in 2–4 weeks. The results described by different surgeons show similar success as the “gold-standard” open procedure around 95% (Table 1).

Management of reoperative pyeloplasty has been performed in six patients with robotic assistance using a transperitoneal approach. The exposure was generally straightforward, although some of the cases required delicate dissection due to the dense periureteral fibrosis. Due to enhanced visualization, this was not any more difficult than with open surgery, and perhaps more efficient. Outcomes with limited follow-up have been satisfactory for this challenging group of patients.

### Vesicoureteral Reflux

#### *Intravesical Approach*

The intravesical approach to ureteral reimplantation using computer assistance follows the same steps as the open, transtrigonal, Cohen procedure, and was first described in 2003[4]. The authors showed good results in a pig model using a system of insufflating the bladder or pneumovesicum. This has been applied to children using conventional laparoscopy[5] and using the robotic system[6].

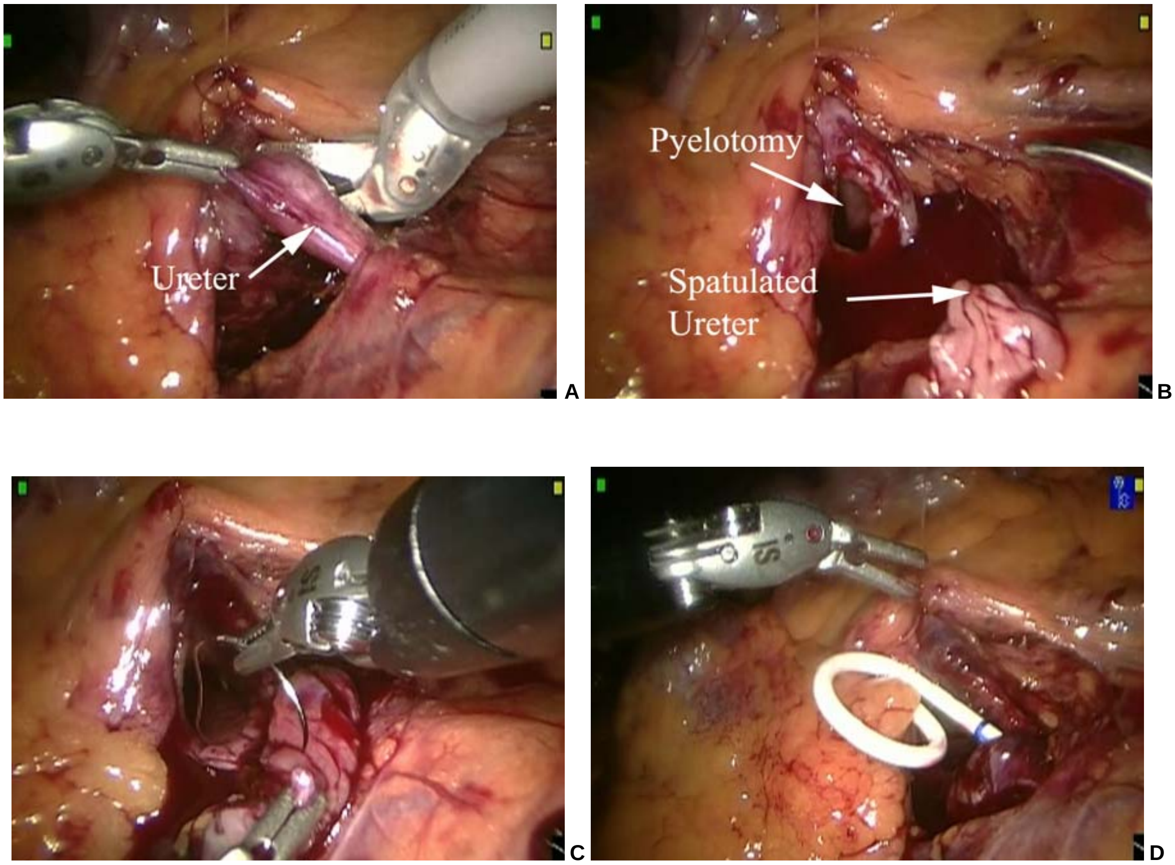

**FIGURE 3.** Robotically assisted laparoscopic pyeloplasty in a school-age child. Using a transmesenteric approach, the pelvis and ureter can be visualized, and the pelvis is stabilized with a hitch stitch. (A) The pelvis is being opened above the UPJ in order to use the pathological segment as a handle during dissection. It is later removed. (B) The proximal lateral ureter has been spatulated to provide a wide anastomotic segment. (C) Anastomosis of the ureter and pelvis is begun, using 5-0 or 6-0 monofilament absorbable suture. (D) A double-J ureteral stent has been placed for postoperative drainage after the back wall of the anastomosis has been completed. (E) Appearance of the completed anastomosis. The peritoneum over the repair will be reapproximated.

**TABLE 1**
**Robotic-Assisted Pyeloplasties: Clinical Outcomes**

| N | Age (years) | Ports | Op. Time (min) | EBL (ml) | Success | Complications | Ref. |
|---|---|---|---|---|---|---|---|
| 33 | 7.9 (range 0.2–19.6) | 3 | 219 | 3 | 97% | One patient required percutaneous drainage. | [7] |
| 7 | 12 (range 6–15) | 4 | 184 | 31.4 | 86% | One patient did not follow-up evaluation. | [8] |
| 15 | 6.7 (range 3.5–16.2) | 4 | 173 | NA | 93.3% | One patient required percutaneous drainage. | [9] |

With the patient in the supine position and the legs apart, the bladder is fully filled with saline solution through the urethra. Using an open technique, the 12-mm camera port is placed in the midline at the bladder dome after a 3-0 Vicryl suture is placed to secure the bladder wall. The working ports, 8 or 5 mm, are positioned midway between the umbilicus and the pubis at the mid-clavicular line. Ports are fixed to the abdomen wall using a stitch which is also used to close the bladder.

Carbon dioxide is insufflated into the bladder to empty it of saline and the robotic device is brought over the patient's feet. A 5 or 8 Fr feeding tube in the urethra is used for suction, positioned as needed by the robotic grasping devices. Similar to the open technique, ureteral dissection starts after placement of a 6-cm length of a 5 Fr feeding tube, sutured in with a 4.0 Vicryl suture to the ureteral meatus. An incision is made around the ureteral meatus leaving a mucosal cuff, which will be used to suture to the vesical mucosal after tunneling. Mobilization of the ureters is done as in the open procedure, taking down the tissue around the ureter using the hook cautery, with care taken to avoid thermal damage (Fig. 4). Prior to submucosal tunnel creation, both ureters are mobilized.

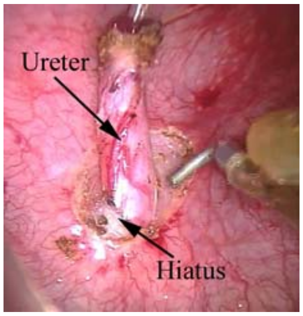

**FIGURE 4.** Ureteral mobilization for reimplantation during a transvesical, robotically assisted, transtrigonal (Cohen) reimplantation. The ureteral vasculature is carefully preserved and can be readily visualized. The mobilization is similar to that performed in open surgery and made efficient by the dexterity of the robotic instruments.

The submucosal tunnels are created by dissecting with scissors from the original meatus to the opposite side of the trigone, and incising the mucosa at the site of the new mucosal hiatus. The articulating capacity of the robotic devices greatly facilitates this maneuver. Anastomosis of the ureters is performed after bringing them through the mucosal tunnel with anchoring sutures of 4.0 monocryl and mucosal sutures of 5.0 monocryl. The mucosa over the hiatus is closed with running 5.0 monocryl. The bladder is filled again with saline solution to check for bleeding and the Foley catheter is positioned.

The working ports are removed first with pneumovesicum evacuation. The bladder-holding stitches are then tied. The endoscope is used to inspect the port sites. Each fascial defect is then closed and the skin is closed with a subcuticular monocryl suture. The bladder catheter is kept in place for 1 day and the patient is discharged home and advised to refrain from play for 1 week.

There are limited data on human applications of this technique[6] and it is our observation that the challenge of efficient access and maintenance of the pneumovesicum has yet to be fully met. In six children (5–15 years old), we have had one conversion to an extravesical, laparoscopic, robotic reimplant and the hospital stay ranged from 2–4 days. One girl had urine leakage from one port site postoperatively secondary to inadequate closure. One patient has persisting low-grade reflux. The advantages over open bilateral, intravesical reimplantation are uncertain, but the excellent visualization and control argues for continued exploration of this approach, as it may be useful for more complex intravesical procedures such as ureterocele excision and bladder neck reconstruction.

### Extravesical Approach

The extravesical approach to laparoscopic correction of reflux was described experimentally in 1993[10] and clinically has been explored by several authors. Most recently, Lakshmanan and Fung[11] have shown efficacy in an extended clinical series, although no comparison with open techniques was carried out. These authors also contend that the known risk of postoperative retention after open, bilateral, extravesical reimplantation is less with laparoscopic methods. We have performed over 30 extravesical, unilateral, antireflux procedures using robotic assistance and this procedure seems to offer a good alternative to open technique with similar success.

With the patient under anesthesia in the supine position, a bladder catheter is placed on the sterile field to permit filing and draining of the bladder. The authors prefer to use an open technique to place the first trocar, the 12-mm camera port, in the umbilicus. The working ports, 8 or 5 mm, are positioned in the mid-clavicular line bilaterally, 1 cm below the umbilical line. Ports are fixed to the abdomen wall using a stitch that is also used to close the fascia. The robotic device is brought over the patient’s feet.

The technique follows the same steps as the open, Lich-Gregoir procedure (Fig. 5). The ureter is dissected free after opening the peritoneum. Approximately 5 cm of ureter is dissected, preserving the peripheral vascularity of the ureter. A hitch stitch may be used to improve the exposure of the posterior bladder wall, attaching to abdominal wall or through it, and to the bladder just above where the tunnel will start. A detrusor trough is made by dissecting the mucosa away from the incised muscular layer of the bladder for about 4 cm. If there is a hole in the mucosa, the authors close it with a 5-0 chromic suture. The bladder muscular wall is then closed over the ureter using 4-0 Vicryl interrupted suture. Care is taken to visualize the ureter throughout this process and to limit tightness to avoid obstruction. The peritoneum is closed in a running fashion and the robot is removed. A bladder catheter is used if there has been significant injury to the mucosa or if there is concern about the patient’s voiding. Most patients are discharged the next morning, although some go home the same day.

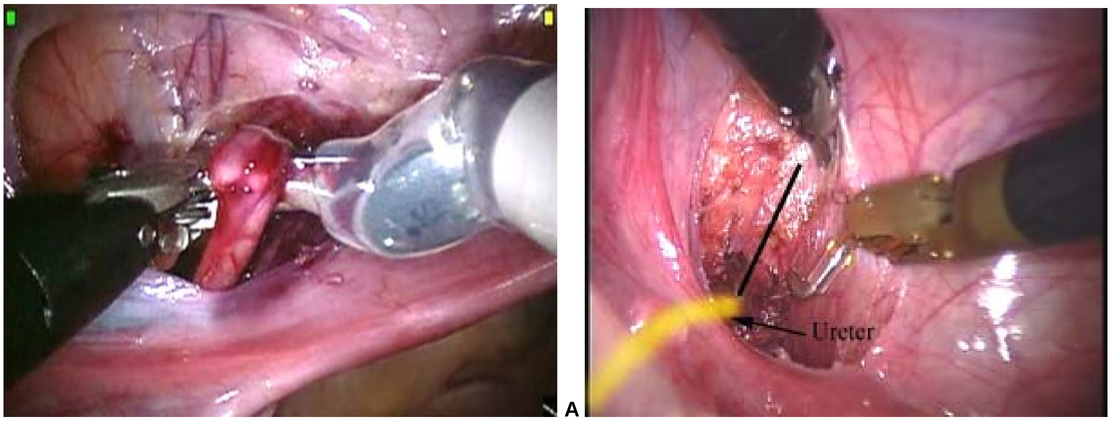

A B

**FIGURE 5.** (A) Appearance of the ureter being mobilized during an extravesical, ureteral reimplantation performed with robotic assistance. The hook cautery is lifting the ureter to permit blunt dissection of the periureteral tissues. (B) The path of the ureter, which has been mobilized, is depicted in yellow. The location of the detrusor trough that will be created is indicated by the line.

Results of this technique using robotic assistance suggest reduced postoperative analgesic requirements, shorter hospital stay, and with more recent cases equal efficacy. Operative times are approaching that for open technique[12].

### CONCLUSION

While still very early in the development phase, the initial results with robotic assistance of laparoendoscopic procedures in pediatric urology has been favorable, with successful completion of a variety of surgical procedures, safely and efficaciously. With improved techniques, tools, and experience, it is likely that these novel methods will become a regular part of the surgical armamentarium of the pediatric urologist.
